# The complete mitochondrial genome of *Inimicus japonicus* and its phylogenetic analysis

**DOI:** 10.1080/23802359.2020.1823270

**Published:** 2020-10-09

**Authors:** Weiye Li, Xiaolin Liu, Jingang Xu, Siyuan Li, Xiaolong Yin, Hulin Xie, Xiaolin Zhang

**Affiliations:** a National Engineering Research Center of Marine Facilities Aquaculture, Zhejiang Ocean University, Zhoushan, China; b Research Institute of Zhoushan, Zhoushan, China; c Marine Science and Technology Fishery College, Zhejiang Ocean University, Zhoushan, China

**Keywords:** *Inimicus japonicus*, mitochondrial genome, phylogenetic relationships

## Abstract

The complete mitochondrial genome was sequenced from the marine teleost fish *Inimicus japonicus*. The genome sequence is 16,830 bp in size and has a base composition of A (29.25%), T (29.01%), C (20.7%), and G (21.03%). Moreover, 13 protein-coding genes (PCGs) encoded 3210 amino acids in total. The phylogenetic analysis showed that *I. japonicus* belongs to Synancejidae family. The complete mitochondrial genome sequences provided here would be useful for further understanding the evolution and conservation genetics of *I. japonicus*.

*Inimicus japonicus* (Scorpaenidae, Scorpaeniformes) is a warm bottom economic fish, distributed in the western tropical and warm temperate parts of the North Pacific, and the East Sea, South Sea, Yellow Sea, Bohai Sea in China (Wang et al. 2013). It is an appropriate species for reef area to enhance and release and the object of sport fishing enthusiasts. In this study, we firstly reported the complete mitochondrial genome of *I. japonicus*, which would help understand the phylogenic relationship of the Scorpaenidae family.

The *I. japonicus* in this study were artificially bred in fisheries research institute of Zhoushan City in Zhujiajian aquaculture base, Zhejiang Province of China. The parent fish were from Ningde Fujian. Samples stored in a refrigerator of −80 °C before sequencing. Illumina PE library was constructed by second generation Illumina Hiseq sequencing technology, and genome mapping was completed by bioinformatics analysis. Approximately, 4982 Mb of raw data and 4734 Mb clean data were obtained, and *de novo* assembled by the SOAP *de novo* software. Moreover, DOGMA (Zhu et al. 2019) and tRNAscan-SE 2.0 (Lowe and Chan 2016) were used to annotate this mitochondrial genome.

The complete mitochondrial genome length of *I. japonicus* was 16,830 bp in length (GenBank accession number MT604162). It consisted of 13 protein-coding genes, 2 rRNA genes, 22 tRNA genes. The overall base composition of the mitogenome is 29.25% for A, 20.7% for C, 21.03% for G and 29.01% for T. Similar to the mitogenomes of other vertebrates, the AT content is higher than the GC content (Song et al. 2020).

To further validate the new sequences and the phylogenetic position of *I. japonicus*, we used the mitochondrial genomes of 26 species which from different genera in Scorpaeniformes to construct the phylogenetic tree (Figure 1). MEGA software was used for constructing a maximum likelihood (ML) tree (Kumar et al. 2016). The tree topologies suggested that *I. japonicus* had the closest relationship with *Synanceis verrucose*. This study will contribute to the phylogenetic classification and the genetic conservation management of *I. japonicus*.

**Figure 1.**
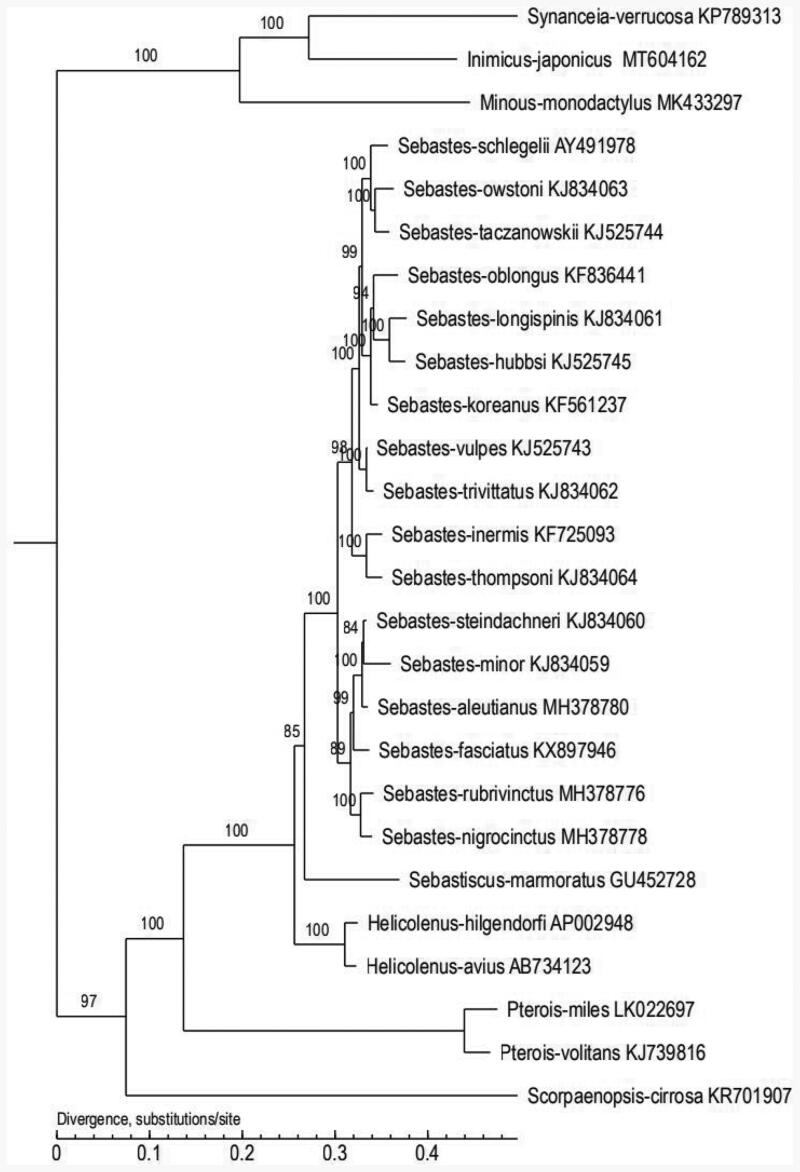
The phylogenetic tree was constructed based on the complete mitochondrial genomes of 26 species which from different genera in Serranidae. The number at each node is the bootstrap probability. The number behind the species name is the GenBank accession number.

## Data Availability

The data that support the findings of this study are openly available in “NCBI” at https://www.ncbi.nlm.nih.gov/, reference number MT604162.
